# The effect of simulation-based education on parental management of fever in children: a quasi-experimental study

**DOI:** 10.1186/s12912-022-00938-7

**Published:** 2022-06-28

**Authors:** Asghar Tavan, Elnaz Monemi, Fateme Keshavarz, Behrooz Kazemi, Monirsadat Nematollahi

**Affiliations:** 1 Health in Disasters and Emergencies Research Center, Institue for futures Studies in Health, Kerman University of Medical science, Kerman, Iran; 2 Pediatric and neonatal intensive care nursing department, Razi school of nursing and midwifery, Kerman University of Medical Sciences, Kerman, Iran; 3 Nursing Research Center, Kerman University of Medical Sciences, Kerman, Iran; 4 Student Research Committee, Kerman University of Medical Science, Kerman, Iran

**Keywords:** Fever, Parents, Simulation-based education, Children

## Abstract

**Background:**

Fever is a sign of illness in children and parents should receive educational interventions based on their needs to provide effective care for children. Simulation-based education provided by nurses for managing children’s fever can help improve the quality of parental care. Accordingly, this study aimed to explore the effectiveness of simulation-based education in the management of children’s fever by parents.

**Methods:**

This quasi-experimental study was conducted using a pretest-posttest design with two groups on 90 parents of children with fever who visited Afzalipour Teaching Hospital in Kerman, Iran. The participants were randomly divided into two groups. The members of the intervention group received simulation-based fever management education and the parents in the control group received routine interventions. A demographic information form and the Parental Fever Management Knowledge and Practice Scale were completed by the participants in both groups before and after the intervention. The collected data were analyzed with SPSS 21 at a significant level of 0.05 (*P* = 0.05).

**Results:**

The results of the study showed that there was a statistically significant difference between the mean scores of fever management knowledge in the intervention group before and after the intervention (30.51 ± 1.50 vs. 54.79 ± 2.55) (*p* < 0.05), while the control group showed no statistically significant difference before and after the intervention (29.81 ± 4.1 vs. 29.95 ± 2.80) (*p* > 0.05). Furthermore, there was a significant difference between the mean scores of fever management practice in the intervention group before and after the intervention (24.32 ± 0.89 vs. 37.51 ± 1.09) (*p* < 0.05). In contrast, the control group showed no statistically significant difference before and after the intervention (23.03 ± 0.90 vs. 21.98 ± 0.02) in terms of fever management practice (*p* > 0.05). The results of the independent samples t-test also showed that the mean scores of fever management knowledge and practice were not significantly different between the two groups before the intervention (*p* > 0.05) while there were significant intergroup differences after the intervention (*p* < 0.05).

**Conclusion:**

The results of the study showed that simulation-based education was effective in improving the parents’ child fever management knowledge and practice. Accordingly, professional care teams can prepare simulation-based education packages to improve parental care at home for children’s fever management.

## Introduction

Fever is one of the most important disease symptoms in children [1] and one of the most common causes for families seeking medical care and attention. Moreover, in many cases, fever is the only reason for family caregivers to refer to medical offices and clinics [2]. The high number of families visiting medical centers due to fever can be attributed to their excessive concern about this symptom [3].

Most parents and caregivers often consider fever to be a disease and prescribe antipyretic drugs to their child before consulting the care team [4]. Parents may also experience abnormal fears when a child develops a fever due to insufficient knowledge and misunderstanding of fever management [5]. However, the level of awareness of families has shown some variations in different studies. For example, de Bont et al. (2014) reported that 88% of mothers had a correct definition of fever [6]. Moreover, Se′ed. et al. (2013) found that about 78% of parents have a correct definition of their child’s body temperature at the time of the fever [7]. In another study, 47% of mothers did not have a correct definition of the standard body temperature and fever [8]. Other studies reported the poor knowledge and practice of parents in measuring fever in their children and using physical methods to reduce fever [9, 10].

Fever in children is better to be managed and cared for at home. However, the low levels of awareness in a majority of caregivers [11, 12] make the management of fever at home difficult in many cases. Besides, insufficient knowledge creates negative effects such as parents’ fear of fever and indirect harm due to improper use of drugs in children with fever [13]. Parental education is essential to reduce the fear of fever in parents and increase their awareness of the proper use of antipyretic drugs [12]. Increasing parents’ awareness and knowledge about managing their child’s fever enhances their self-confidence and as a result, they perform better in care [14].

Hermalinda positively evaluated the effect of implementing an education program on parental behavior in controlling pediatric fever in Indonesia [15]. Moreover, Broome et al. and Chang et al., in separate studies, showed improvements in parental performance in enhancing their children’s fever management by implementing educational models [16, 17]. Scenario-based simulation education is one of the training methods that by placing people in the real environment, can lead to the acquisition of expected skills and competencies by the learner [18]. Parents of children with fever need to use effective teaching methods to properly manage their children’s fever because they still have problems in managing their children’s fever by using the conventional training methods [2]. Accordingly, the present study aimed to explore the effectiveness of simulation-based education in the management of children’s fever by parents.

## Materials and methods

### Research design and setting

This study was conducted using a quasi-experimental design on parents of children with fever who visited Afzalipour Hospital in Kerman, Iran.

### Participants and sampling

Following a pilot study and with 80% power and 95% confidence interval, the sample size was estimated to be 100 persons and considering the 20% dropout probability, 50 parents were selected for each group. Sample size determined by Koopak formula using G*Power version 3.1.9.7.

The participants were 100 parents of children aged 3 months to 14 years. Parents were selected using convenience sampling and were then randomly divided into control and intervention groups by numerical table.

Five parents in each group were excluded from the study due to discharge before completing the empowerment intervention. Finally, 90 persons were included in the study (45 people in each group) and the sample dropout rate was 10%.

The inclusion criteria were Have a hospitalized child with an acute illness with fever such as gastro enteritis, acute media otitis, upper respiratory infection, being able to understand and speak Persian, parents have a good mental health and the ability to understand educational content.

For randomization based on the numbers in the table, the first two persons were placed in the intervention group and the next two people were placed in the control group, and this process was repeated until sampling was completed.

### Data collection

The data in this study were collected using a demographic information form and the Parental Fever Management Knowledge and Practice Scale. The demographic information form measured the participants’ age, relationship with the child, marital status, education, occupation, the child’s sex, and the child’s age. The Parental Fever Management Knowledge and Practice Scale contained 31 items and two sections, measuring family caregivers’ knowledge and skills for managing children’s fever. The section measuring fever management knowledge contained 10 items addressing the conditions requiring emergency visits to the doctor (with a score of 10 to 30), 7 items specifying the reasons for emergency visits to the doctor (with a score of 7 to 21), and 5 items measuring caregivers’ understanding of how to improve the child’s condition and reduce the child’s temperature (with a score of 5 to 15). All items in the fever management knowledge subscale were scored as *true* (3), *no comment* (2), and *false* (1). The overall score on this subscale ranged from 22 to 66, with higher scores indicating higher levels of knowledge. Furthermore, the fever management practice subscale contained 9 items scored on a five-point Likert scale ranging from always (5), often (4), sometimes (3), rarely (2), and never (1), with a total score ranging from 9 to 45 and the higher the score, the better the caregiver skills in managing the fever.

The Parental Fever Management Knowledge and Practice Scale was a researcher-made instrument developed based on a review of previous studies in the literature. For validity of the Scale, the scale was reviewed by ten faculty members and revised based on their feedback. The Cronbach’s alpha for the scale was estimated as 0.89 using the test-retest method, showing the acceptable reliability of the scale.

### Data analysis

The collected data were analyzed using descriptive (frequency, percentage, mean, and standard deviation) and inferential statistics. According to the Kolmogorov– Smirnov test results, the data in this study followed a normal distribution. Thus, parametric tests were used. Furthermore, independent samples t-test was employed to compare the mean scores of fever management knowledge and practice between the intervention and control groups before and after the intervention. The paired samples t-test was also used to compare the mean scores of fever management knowledge and practice in each group before and after the intervention. *P*-values less than 0.05 (*p* < 0.05) were considered statistically significant.

### Procedure

After obtaining the necessary permits and the code of ethics for the research project, the researcher visited the pediatric ward of Afzalipour Hospital. After explaining the objectives of the study to the participants, written consent was obtained from them. The parents were reassured that attending or not attending the study would not affect the treatment of their children in the ward.

Before conducting the intervention, the participants in the two groups first completed the items in the demographic information form and the Parental Fever Management Knowledge and Practice Scale. The training intervention started on the second day of the child’s hospitalization for the participants in the intervention group. The reason for starting the intervention on this day was to check the child’s condition, diagnose the disease, perform required tests, transfer the child from the emergency department to the pediatric ward, and ensure parental and child comfort. The researcher who held a Ph.D. degree in pediatric nursing conducted the intervention following the training protocol (definition of fever, ways to control fever, how to use different types of thermometers, ways to reduce fever, cases required seeing a doctor immediately, cases required emergency visits to a doctor, complications of fever) (Table 1) in four one-hour sessions (in groups of 2 to 3 people) in a simulated environment located in the pediatric ward. The training sessions were held using equipment such as boards, teaching aids, children’s models, various thermometers, and a set of fever control devices.

**Table 1:** Contents trained in each session

Sessions	Trained contents
First session	Fever definition Importance of fever management Parents’ stress in fever management Necessary items to refer a doctor immediately Group discussion about trained contents
Second session	How to measure a child’s temperature Work with a thermometer and read the temperature Practice on modeling
Third session	Applying appropriate Interventions for lowering the temperature based on the child’s temperature Appropriate drug treatments Practicing based on scenarios
Fourth session	Common Mistakes in Controlling Pediatric Fever Summarize the whole content and repeat the key points of the fever control

The training sessions were held in the evening shifts as the ward was less crowded and the father or other family members attended the child’s bedside so that the mother could attend the sessions for an hour without worry. At the beginning of each session, the researcher talked about the content of the session for 45 minutes, and at the end of each session, she asked the mothers some questions about the instructional materials provided for 14 mins. If the instructions required the parents’ practice on the child’s model, the participants could put the instructions into practice on the model. In the third and fourth sessions, the researcher presented some pre-prepared scenarios, and the parents were required to provide care according to the scenarios. These scenarios were developed based on the biographies of three children who had different illnesses and had a fever as a sign of their illness. The training sessions were held in the form of group discussions and the participants could ask their questions (if any). This process continued until the fourth day of hospitalization. During these 4 days, in addition to the face-to-face training intervention, a telephone number was given to the parents to ask their questions at specific hours of the day [16–18]. Furthermore, whenever the child needed care for fever management during the intervention, the parents performed the relevant care under the supervision of the pediatrician, and this process continued until discharge. At the time of discharge, a booklet containing the instructed materials was given to each parent. At the end of the first week after discharge, the Parental Fever Management Knowledge and Practice Scale was completed for the participants by telephone interviews. Moreover, if one of the parents failed to attend the training sessions, he/she would receive individual training.

The participants in the control group received standard interventions including routine care and training in the ward. Pediatric fever control educational interventions were not performed in the ward in a systematic way or according to a specific protocol. These parents trained by pediatric nurses in the ward. The participants in the control group also completed the Parental Fever Management Knowledge and Practice Scale at the end of the first week. At the end of the study, the parents of the children in the control group were given a fever control training booklet. To prevent any contact between the parents in the control and intervention groups, they were selected from the two different pediatric wards that were similar in terms of equipment, facilities, and trained nurses.

## Results

The data in this study showed that the participants in the two control and intervention groups had no significant differences in terms of demographic characteristics such as parents’ age, marital status, education, occupation, the child’s age, and the child’s gender (Table 2).

**Table 2:** Demographic characteristics in parents in the control and intervention groups

Variable		Control group		Target group		Chi-square test
		N	Percent	N	Percent	
Age	Under 20y	2	4.4	1	2.2	*P* = 0.8* χ2 = 0.35
	20-30y	20	44.4	21	46.7	
	Upper 30y	23	51.1	23	51.1	
	Total	45	100	45	100	
Marital status	single	2	4.4	2	4.4	*P* = 0.07* χ2 = 5.3
	married	38	84.5	40	88.9	
	divorced	5	11.1	3	6.7	
	Total	45	100	45	100	*P* = 0.52* χ2 = 1.27
Education	Under diploma	18	40	13	28.9	
	graduated	17	37.8	21	46.7	
	Post graduated	10	22.2	11	24.4	
	Total	45	100	45	100	
Job	housewife	35	77.8	39	86.7	*P* = 0.33* χ2 = 2.2
	government job	6	13.3	2	4.4	
	private job	4	8.9	4	8.9	
	Total	45	100	45	100	*P* = 0.27* χ2 = 1.19
Children sex	boy	26	57.8	31	68.9	
	girl	19	42.2	14	31.1	
	Total	45	100	45	100	
Children age	under 1y	15	33.3	12	26.7	*P* = 0.26* χ2 = 3.9
	1-3y	17	37.8	14	31.1	
	3-5y	4	8.9	11	24.4	
	Upper 5y	9	20	8	17.8	
	Total	45	100	45	100	

Qui square*

Besides, the results of paired samples t-test showed that there was a statistically significant difference between the mean scores of fever management knowledge in the intervention group before and after the intervention (30.51 ± 1.50 vs. 54.79 ± 2.55) (*p* = 0.001), while the control group showed no statistically significant difference in terms of fever management knowledge before and after the intervention (29.81 ± 4.1 vs. 29.95 ± 2.80) (*p* = 0.123). Additionally, the results of the independent samples t-test indicated a significant difference between the mean scores of fever management practice in the intervention group before and after the intervention (24.32 ± 0.89 vs. 37.51 ± 1.09) (*p* = 0.005). However, the control group showed no statistically significant difference before and after the intervention (23.03 ± 0.90 vs. 21.98 ± 0.02) in terms of fever management practice (*p* = 0.45). The results of the independent samples t-test also indicated no significant differences between the two groups in terms of fever management knowledge (*p* = 0.21), and practice (*p* = 0.16) before the intervention while there were significant between two groups differences in the field of knowledge (*p* = 0.001) and performance (*p* = 0.002) after the intervention. These findings indicated that the implementation of the fever management training program was effective in improving fever management knowledge and practice of family caregivers of children with fever (Table 3).

**Table 3:** Comparison of the fever knowledge and performance scale in parents in the control and the intervention group before and after

	Variables	Group	Before	After	*p*-value
**Knowledge**	**emergency refered** to doctor (10–30)	Control Intervention	13.56 ± 2.45 13.06 ± 0.78	13.1 ± 1.05 23.89 ± 1.24	0.42* 0.00*
	Between two groups	*p*-value	0.12**	0.002**	
	referred to a doctor when the child has a fever (7–21)	Control Intervention	10.67 ± 1.2 10.91 ± 0.22	10.80 ± 0.7 17.67 ± 1.22	0.74* 0.01*
	Between two groups	*p*-value	0.23**	0.01**	
	**Symptoms for understanding decreasing temperature (5–15)**	Control Intervention	6.08 ± 0.87 6.54 ± 0.56	6.05 ± 1.12 13.23 ± 0.09	*P* = 0.123* P0.001*
	Between two groups	*p*-value	0.14**	0.015**	
	**Total Knowledge)22–66)**	Control Intervention	29.81 ± 4.1 30.51 ± 1.50	29.95 ± 2.80 54.79 ± 2.55	*P* = 0.23* *P* = 0.001*
	Between two groups	*p*-value	0.21**	0.001**	
	**Performance**	Control Intervention	23.03 ± 0.90 24.32 ± 0.89	21.98 ± 0.02 37.51 ± 1.09	0.48* 0.005*
	Between two groups	*p*-value	0.16**	0.002**	

*paired t-test

**independent t-test

## Discussion

The present study examined the effectiveness of simulation-based education in the management of children’s fever by parents. The results indicated that simulation-based education improved the fever management knowledge and practice in the parents who participated in the intervention program (*p* < 0.05). However, the participants in the control group who received only routine care showed no significant improvement in terms of fever management knowledge and practice (*p* < 0.05). Simulation-based education not only increases learners’ knowledge but also improves their skills [18]. In this training method, learners can acquire problem-solving skills in the simulated environment and optimally put what they have learned into practice in the real environment [19]. Several studies indicated the positive effect of parental education on the management of their children’s fever. For example, Herman and Nurshal (2017) showed that the implementation of a parent education program in Indonesia increased parents’ knowledge, attitudes, and practice in controlling their children’s fever. The researchers provided the necessary training for fever management to parents in 30-minute sessions using lectures [15]. Although this study highlighted the effectiveness of education, it differed from the current study in terms of parental education. Moreover, Thota et al. showed that increasing parents’ knowledge in fever management reduced parental errors in the use of antipyretics and antibiotics for children [10]. In line with the results of the current study, other studies highlighted the effectiveness of educational programs in parental fever management [20, 21]. Alqudah et al. (2014) used DVDs and brochures to train parents to manage their children’s fever, which was quite different from the method adopted in the present study. Nevertheless, Alqudah et al. (2014) considered the use of DVDs to educate parents a kind of educational innovation [20]. They showed visual and written training modules were more effective than written training protocols. The authors also suggested that nurses use online methods for continuing education in families, because the continuation of education can have positive outcomes.

Broome et al. (2003) showed that the use of an educational model improved the performance of parents in improving their children’s fever management. The authors trained the parents using brochures and educational videos [16]. In line with the results of the current study, Chang et al. (2016) showed that simulation-based education increased information and motivation and enhanced the behavior of Taiwanese parents in managing their children’s fever [17]. This study was similar to the current study as they both focused on simulation-based education. However, both simulation and group discussions were used in the present study. In addition to practice sessions in the simulated environment, family caregivers used the learned skills for controlling their children’s fever. In fact, one of the strengths of the present study was the use of designed scenarios and parental practice in a real clinical setting at the child’s bedside since the use of the instructions for real patients would make the effect of the instructions last for a longer time.

The results of this study showed that the participants in the control group showed no statistically significant difference in terms of fever management knowledge and practice before and after the intervention (*p* > 0.05). Since the members of the control group did not receive structured training on fever control methods by nurses and physicians, no statistically significant improvement was observed in their fever management knowledge and practice.

Although scenario-based education has its advantages, it also has some limitations. For instance, the successful implementation of this educational method requires experienced teachers [22] and also prior needs analysis so that an effective training program can be developed for the learners. However, parents of children should have access to reliable scientific information resources so that they can effectively control the children’s fever. Thus, healthcare professionals should provide parents with reliable and consistent information about fever control to help them with the management and care of the child. Furthermore, nurses and physicians should evaluate parents’ knowledge and practice in fever management so that they can prepare simulation-based training programs based on parents’ needs.

### Limitations

The child’s restlessness and parents’ fatigue and impatience could affect their responses to the questionnaires and motivation to participate in the study. The researcher tried to overcome these problems by explaining the objectives of the study to participants. Besides, the interventions were performed when the parents had enough time and patience as well as mental preparation. This also helped the parents complete the questionnaires without stress. Another limitation of this study was the absence of a long-term follow-up to examine the retention effects of the training program on the parents. Furthermore, the questionnaires were completed after the intervention through phone calls with the participants, which could affect the accuracy of the data. Another limitation of the study was that the study was performed on parents of a medical center, and perhaps if it was performed on parents of children with chronic diseases and more numbers, different results would be obtained.

## Conclusion

The results of the present study showed that the simulation-based education program improved home caregivers’ knowledge and skills in managing their children’s fever. Implementing fever management simulation training for parents and the use of common training methods such as training videos and brochures can increase the effectiveness of training programs. Thus, simulation-based family-centered training programs can be developed to help parents properly manage children’s fever and provide care for them at home. Moreover, future studies can examine the effect of implementing simulation-based training on parents’ fever management skills in the time before emergency visits to medical offices and hospitals.

## Data Availability

The datasets used and analyzed during the current study are available from the corresponding author on reasonable request.
